# Initiation and modulation of Tau protein phase separation by the drug suramin

**DOI:** 10.1038/s41598-023-29846-9

**Published:** 2023-03-09

**Authors:** Prabhu Rajaiah Prince, Janine Hochmair, Hévila Brognaro, Susanna Gevorgyan, Maximilian Franck, Robin Schubert, Kristina Lorenzen, Selin Yazici, Eckhard Mandelkow, Susanne Wegmann, Christian Betzel

**Affiliations:** 1 Institute for Biochemistry and Molecular Biology, Laboratory for Structural Biology of Infection and Inflammation, University of Hamburg, c/o DESY, 22603 Hamburg, Germany; 2 The Hamburg Centre for Ultrafast Imaging (CUI), University of Hamburg, 2276 Hamburg, Germany; 3 German Center for Neurodegenerative Diseases (DZNE), 10117 Berlin, Germany; 4 German Center for Neurodegenerative Diseases (DZNE), 53127 Bonn, Germany; 5 Department of Neurodegenerative Diseases and Gerontopsychiatry, Univ. Clinic, Bonn, 53127 Bonn, Germany; 6 European XFEL GmbH, 22869 Schenefeld, Germany

**Keywords:** Chemical biology, Neuroscience

## Abstract

Tau is an intrinsically disordered neuronal protein in the central nervous system. Aggregated Tau is the main component of neurofibrillary tangles observed in Alzheimer’s disease. In vitro, Tau aggregation can be triggered by polyanionic co-factors, like RNA or heparin. At different concentration ratios, the same polyanions can induce Tau condensates via liquid–liquid phase separation (LLPS), which over time develop pathological aggregation seeding potential. Data obtained by time resolved Dynamic Light Scattering experiments (trDLS), light and electron microscopy show that intermolecular electrostatic interactions between Tau and the negatively charged drug suramin induce Tau condensation and compete with the interactions driving and stabilizing the formation of Tau:heparin and Tau:RNA coacervates, thus, reducing their potential to induce cellular Tau aggregation. Tau:suramin condensates do not seed Tau aggregation in a HEK cell model for Tau aggregation, even after extended incubation. These observations indicate that electrostatically driven Tau condensation can occur without pathological aggregation when initiated by small anionic molecules. Our results provide a novel avenue for therapeutic intervention of aberrant Tau phase separation, utilizing small anionic compounds.

## Introduction

**Figure 1:**
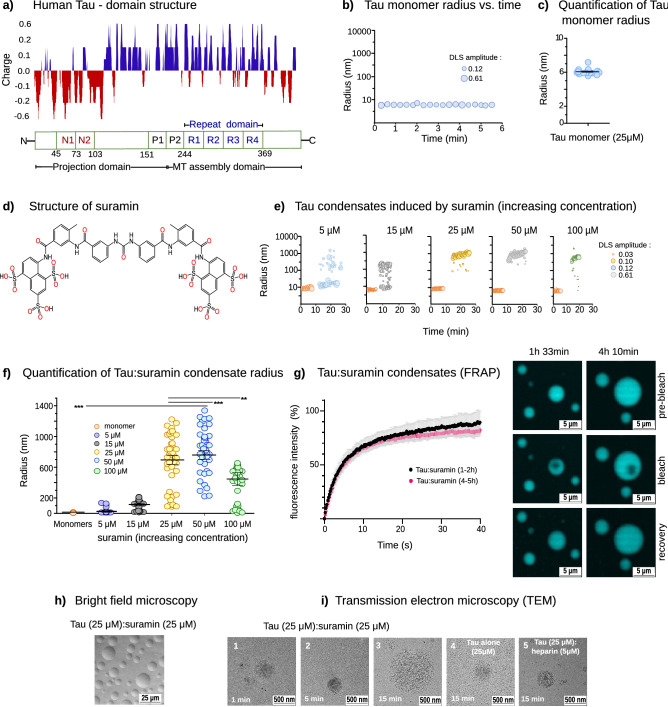
(**a**) Domain structure of human Tau (2N4R isoform), and charge distribution at pH 7.4. MT (Microtubule assembly domain), Projections domain and Repeat domain. (**b**) Representative time resolved DLS (trDLS) detection of 25 μM Tau monomers in solution. The DLS radius plot represents radii of Tau monomers in solution (~ 6 to 8 nm) (x-axis) over time (y-axis). The radii dimension corresponds to DLS amplitudes, which are proportional to the intensity of scattered light of the respective particles. (**c**) Quantification of Tau monomer radius. Radii of monomers were calculated from three independent experiments representing more than 15 data points compliant with the autocorrelation, and are shown as mean ± SEM. (**d**) Structure of suramin (prepared by ACD/ChemSketch 12.0). The molecule is elongated and 8.7 nm apart (PDB 3bf6) and carries 6 sulfonate groups (neg. charge − 6), 3 at each end. (**e**) Tau condensates with suramin at increasing concentration. Representative trDLS data of 25 μM Tau with the addition of 5, 15, 25, 50, 100 μM suramin after ~ 10 min. interval. Radius plot show an increase of condensate dimensions upon suramin addition in low salt buffer. The condensates radii dimensions correspond to DLS amplitudes, which are proportional to the intensity of scattered light of the respective particles. (**f**) Quantification of Tau:suramin condensates at increasing suramin concentration. Tau:suramin condensate radius were calculated from 2 independent experiments, representing more than 20 data points, compliant with the autocorrelation and are shown as mean ± SEM. The data have been compared by one-way ANOVA with Tukey test for multiple comparison. *P < 0.05, **P < 0.01, ***P < 0.001, ****P < 0.0001. (**g**) FRAP measurements of Tau (25 μM): suramin (25 μM) condensates 1–2 h and 4–5 h after LLPS induction in 25 mM HEPES pH 7.4, 10mM NaCl, 1 mM DTT. Representative images of FRAP recovery are shown on the right. Scale bars correspond to 5 μm. Data shown as mean ± SD, n = 20–22 condensates per condition and time point. (**h**) Brightfield microscopy of Tau condensates formed upon equimolar addition of suramin (25 μM). Scale bar corresponds to 25 μm. (**i**) Transmission electron microscopy (TEM) micrographs of Tau:suramin condensates at 1, 5 and 15 min after suramin (25 μM) addition to Tau (25 μM) in low salt buffer. The Tau:suramin condensates were uniformly globular in shape and ~ 500 to 1000 nm in dimensions ROI (region of interest) (**i.1–3**). The Tau:suramin condensates were positively stained (2% Uranyl acetate). The samples (**i.4–5**) show condensates after 15 min incubation of Tau alone (**i.4**) and with heparin (**i.5**) as control. Tau without suramin resembles mesoscopic Tau clusters with globular shape and have a dimension in the range of ~ 100 nm. Scale bar corresponds to 500 nm.

The axonal microtubule associated protein Tau (MAPT) is an intrinsically disordered protein for which a growing number of interactions and functions are defined beyond its canonical role of microtubule (MT) binding^1,2^. Tau misfolding and aggregation in Alzheimer’s disease (AD) and other neurodegenerative diseases point out the relevance of understanding the structural dynamics of Tau and its interaction partners in more detail. Data from numerous *in vivo* and *in vitro* studies attributed Tau’s aggregation to Tau mutations^3^, increased phosphorylation^4^, and interactions with polymeric polyanions (e.g., glycosaminoglycans, heparin)^5^ and RNA^6–10^. Interestingly, Tau interactions with polyanions *in vitro* can induce both Tau aggregation into amyloid fibrils as well as Tau LLPS^11,12^, whereby the ratio of Tau protein to polyanion and the polyanion chain length are essential for the outcome. In the case of LLPS, polyanionic polymers co-condensate with Tau under charge matching conditions into liquid dense condensates (= coacervation)^11,13,14^. Whether also small anionic compounds can induce Tau LLPS is not known. Tau coacervation with polyanionic polymers is governed by intermolecular electrostatic interactions between the negatively charged polymers and positively charged Tau domains, i.e., the C-terminal 2/3 of Tau (Fig. 1a). Recombinant Tau (2N4R isoform) has a net charge of + 2.9 at pH 7.4 (http://protcalc.sourceforge.net/cgi-bin/protcalc). Post-translational modifications (PTMs) that change the charge state of Tau, such as acetylation^15^ and phosphorylation^16^, seem to inhibit Tau coacervation with RNA. Recently, we showed that extended incubation of Tau coacervates leads to the nucleation of Tau species that can seed the aggregation of Tau in cells^16^. The evolution of seeding competent protein species from liquid-condensed protein phases was suggested to play a role for the pathological activity and aggregation of FUS, hnRNPA1, and TDP-43 in amyotrophic lateral sclerosis^17,18^, for alpha-synuclein in Parkinson’s disease^19^, and for Tau in AD and FTD^20,21^. Inhibiting the development of aggregates from condensates of these proteins harbors an interesting therapeutic potential in the treatment of these protein aggregation diseases. Here, we explore whether a small anionic compound could interfere with the formation of seeding potent Tau species in Tau condensates as a potential new therapeutic approach in AD and related diseases. To test this idea, we used the anionic compound suramin, a well-studied antiparasitic drug applied for treating African sleeping sickness^22^. Suramin is further used as a microfilaricide to treat River blindness (onchocerciasis)^23^ and also demonstrates antiviral activity towards HIV and SARS-CoV-2 via the inhibition of RNA polymerases^24^. In addition, it was reported that low concentrations of suramin can inhibit the aggregation of the human islet amyloid polypeptide (IAPP)^25^, and of seminal amyloid fibrils, a prime target for HIV-1 entry^26,27^. For Tau, small molecule modulators of aggregation have been studied in the past, mostly with the aim of blocking the formation of β-structure^28^, but suramin, to our knowledge, has not been tested for this activity.

Our study shows that suramin can modulate and reverse Tau’s coacervation with heparin and RNA, and further reduces the seeding potential of aged Tau coacervates in a cell model of Tau aggregation. Considering that aged Tau condensates with seeding potential could be the initial cause for Tau aggregation in the brain, our data obtained highlight the potential of suramin—and potentially other small anionic compounds—in reducing Tau pathogenicity at an early stage of the disease.

## Results

### Suramin induces Tau condensation

To analyze whether the small anionic compound suramin can induce Tau condensation, like polyanionic polymers (eg. RNA, heparin), we performed time-resolved dynamic light scattering (trDLS), a method to monitor the particle distribution in a solution over time^15,29^ and therefore suited to characterize protein phase separation in initial stages of the condensation process. DLS has previously been used to analyze the liquid–liquid phase separation of intrinsically disordered proteins^30,31^, and by us to monitor Tau condensation^16^. First, we measured the particle size distribution of recombinant human full-length Tau (441 aa; 2N4R isoform; 25 µM in 25 mM HEPES, 10 mM NaCl, 1 mM DTT pH7.4; Fig. 1a) by trDLS for 5 to 10 min. These measurements confirmed the presence of Tau monomers with a hydrodynamic radius (R_h_) of 6 ± 0.08 nm (Fig. 1b,c). These Tau monomer dimensions were in good agreement with previously published SAXS data and our recent SAXS experiment which provided a Rg value of 5 ± 0.06 nm^30,31^. At higher concentrations (50 µM), Tau formed spontaneous mesoscopic "self-clusters" (R_h_: ~ 100 nm) and had larger monomer radius (R_h_: ~ 10 nm; Supplemental Fig. S1a,b). We previously reported similar dimensions for Tau monomers and mesoscale clusters obtained by trDLS^15,16^.

Suramin is a negatively charged compound that carries six sulphonate groups (2 triplets spaced 8.7 nm apart; PDB 3bf6; net charge: − 6 at pH 7.4^32^; Fig. 1d). To analyze whether suramin can induce Tau LLPS at charge matching conditions—in analogy to heparin and RNA^16^, we added increasing concentrations of suramin (5, 15, 25, 50 and 100 µM) to Tau (25 µM) in the DLS cuvette and monitored the evolving particle dimensions (Fig. 1e,f).

Immediately after the addition of suramin, a few Tau:suramin condensates began to form (R_h,t=10 min_: ~ 25 nm) with 5 µM suramin, and more robust condensate formation was observed with 15 µM suramin (R_h,t=10 min_: ~ 100 nm) (Fig. 1e,f). The size of condensates with 25 µM, 50 µM, and 100 µM suramin were R_h_: ~ 600 nm, R_h_: ~ 800 nm, and R_h_: ~ 400 nm, respectively. Light microscopy confirmed that these larger particles were Tau condensates (Fig. 1h) with liquid-like fluorescence after photo bleaching (Fig. 1g). Notably, Tau:suramin condensate dimensions observed by microscopy (radius ~ 5 to 25 µm; at 25 μM concentrations) were larger than that observed by DLS, likely because of their attachment and wetting of the supporting glass surface, a Tau condensate behavior typically observed for Tau:polyanion coacervates^16^.

We used transmission electron microscopy (TEM) as another complimentary method to visualize Tau:suramin condensates (Fig. 1i). Tau:suramin condensates, negatively stained with uranyl acetate, appeared as circular protein "patches" on the EM grid, suggesting condensate sizes (radius ~ 200 to 500 nm) like those we have observed by DLS. For comparison, samples after 15 min incubation of Tau:heparin were visualized applying TEM. The condensates are globular in shape with radii of ~ 200 to 500 nm (Tau:heparin). Notably, Tau self-clusters identified by DLS could also be observed by TEM.

In summary, these experiments showed that the addition of suramin induces Tau:suramin condensation, whereby the dimension (radius) of Tau:suramin condensates increased with suramin concentrations and peaked at Tau-suramin charge matching conditions (negative charges provided by the polyanion equal the number of positive charges provided by Tau).

### Tau:suramin condensation depends on weak electrostatic interactions

**Figure 2:**
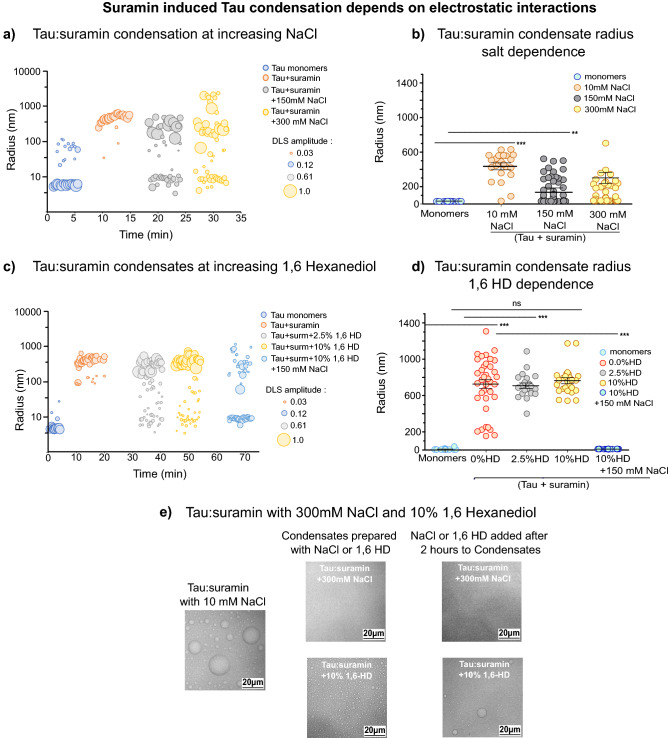
Suramin induced Tau condensation depends on electrostatic interactions. (**a**) Representative trDLS of Tau condensation in low salt buffer, with gradient increase of NaCl. trDLS of 25 μM Tau with addition of 25 μM suramin after ~ 7 to 10 min, followed by addition of 150 mM NaCl (at ~ 17 min), and to 300 mM NaCl (at ~ 25 min). The condensates start to dissolve into monomers and larger aggregates upon increasing the ion concentration to 150 mM or higher. Data point radius sizes correspond to DLS amplitudes, which are proportional to the intensity of scattered light of the respective particles. (**b**) Quantification of salt dependent Tau:suramin condensate radius. Radii of condensates were calculated from 2 independent experiments representing more than 20 data points and are shown as mean ± SEM. Data have been compared by one-way ANOVA with Tukey test for multiple comparison. *P < 0.05, **P < 0.01, ***P < 0.001, ****P < 0.0001. (**c**) Representative trDLS of Tau condensation at increasing 1, 6 Hexanediol (1,6 HD). trDLS of 25 μM Tau with addition of 25 μM suramin after ~ 7 to 10 min, followed by addition of 2.5% 1,6-HD (at ~ 30 min) and 10% (at ~ 45 min), and upon increasing the NaCl concentration to 150 mM (at ~ 80 min). The condensates are moderately stable in 1,6 HD but start to dissolve into monomers and larger aggregates upon increasing the ion concentration to 150 mM NaCl. Data points of the radius dimension correspond to DLS amplitudes, which are proportional to the intensity of scattered light of the respective particles. (**d**) Quantification of 1, 6 Hexanediol (1,6 HD) dependent Tau:suramin condensate radii. Radii of condensates were calculated from 2 independent experiments, representing more than 20 data points and are shown as mean ± SEM. Data have been compared by one-way ANOVA with Tukey test for multiple comparison. *P < 0.05, **P < 0.01, ***P < 0.001, ****P < 0.0001. (**e**) Brightfield microscopy of Tau (25 μM):suramin (25 μM) condensates in low salt (10 mM NaCl) and high salt (300 mM NaCl) conditions or with 10% 1,6 Hexanediol; buffer (25 mM HEPES pH 7.4, 10mM NaCl, 1 mM DTT). Scale bar corresponds to 20 μm.

Tau condensation induced by polyanionic polymers, like RNA and heparin, is based on coacervation of the negatively charged polymers with the positively charged microtubule-binding of Tau^11,12,29^. Interestingly, Tau can also "self-coacervate" based on electrostatic interactions between its negatively charged N-terminal region (up to residue ~ 120) and the positively charged part of the protein (Fig. 1a). Since suramin is a negatively charged molecule, we hypothesized that suramin-induced Tau (net charge: + 2.9 at pH 7.4) condensation depends on electrostatic interactions, like complex coacervation of Tau with RNA and heparin. Since electrostatic interactions can be weakened through shielding by counter ions in solution^32–34^, we prepared Tau:suramin condensates first at low salt conditions in 10 mM NaCl (R_h_: ~ 400 nm) and then stepwise increased the NaCl concentration in the buffer to 150 mM NaCl, and then to 300 mM NaCl. Already at 150 mM NaCl, Tau monomers re-appeared along with polydisperse condensates (R_h_: ~ 100 nm; Fig. 2a,b), indicating that increasing the salt concentration to 150 mM or higher destabilizes preformed Tau:suramin condensates. We confirmed these observations by light microscopy (Fig. 2e), where Tau:suramin condensates disappeared upon increasing the NaCl concentration from 10 to 300 mM and did not form at all in the presence of 300 mM NaCl at start. These observations indicate that suramin induced Tau condensation is driven by electrostatic interactions. Previously it was shown that Tau self-coaceravates^12,13,16^ also decrease in size with increasing salt concentration. Whether Tau:suramin condensation depends on coacervation of suramin with Tau could not be analyzed due to the lack of fluorescently labeled suramin.

We next assessed whether hydrophobic interactions are also vital in Tau:suramin condensate formation. The aliphatic alcohol 1,6-hexanediol (1,6-HD) can inhibit protein LLPS by disrupting hydrophobic interactions in condensates, which has been shown, for example, for Tau condensates induced by the molecular crowder PEG^21^. We first prepared Tau:suramin condensates at 10 mM NaCl (R_h_: ~ 700 nm) and then stepwise increased the concentration of 1,6-HD from 2.5% to 10%. At both 2.5% and 10% 1,6-HD, the condensates remained mostly intact (2.5% 1,6-HD: R_h_: ~ 600 nm; 10% 1,6-HD: R_h_: ~ 700 nm), with only few monomers (10% 1,6-HD: R_h_: ~ 10 nm) (Fig. 2c–e). This suggested that hydrophobic interactions had a minor contribution to the integrity of Tau:suramin condensates. Interestingly, when increasing the salt concentration to 150 mM NaCl at 10% 1,6-HD, the condensates immediately dissolved into Tau monomers (< R_h_: ~ 10 nm), confirming that ionic interactions are driving and stabilizing Tau:suramin condensates (Fig. 2c–e).

To evaluate interaction strength between suramin and Tau, we determined their binding affinity in solution by microscale thermophoresis (MST)^35^. Titration of suramin to fluorescently labelled Tau induced a change in fluorescence that corresponded to a moderate Kd of ~ 17 µM (Supplemental Fig. S2a). Notably, suramin also exhibits moderate to high binding (Kd) with many other protein targets^26,36–39^. However, in this study, the weak electrostatic interactions between suramin and Tau are in line with the concept that multivalent molecular interactions are the main drivers of protein LLPS.

### Suramin disrupts Tau:heparin condensates

**Figure 3:**
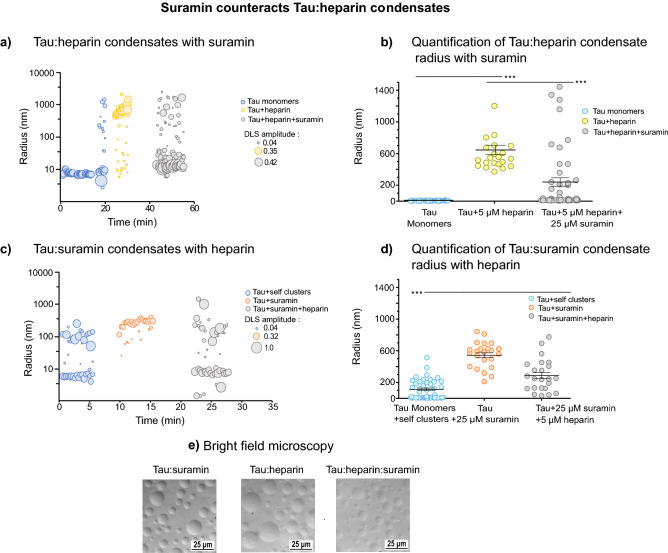
Suramin counteracts Tau:heparin condensates. (**a**) Tau:heparin condensates with suramin. Representative size distribution of Tau:heparin condensates at 25 μM Tau in low salt (10 mM NaCl) buffer with the addition of 5 μM heparin after ~ 20 min, followed by addition of 25 μM suramin (at ~ 40 min). trDLS detects Tau:heparin condensates (radii ~ 500 nm) and with addition of 25 μM suramin after ~ 10 min. results in the generation of smaller Tau:heparin condensates with a radii of ~ 200 nm. Data point radii correspond to DLS amplitudes, which are proportional to the intensity of scattered light of the respective particles. (**b**) Quantification of Tau:heparin condensate radii with suramin. Radii of condensates were calculated from 2 to 3 independent experiments, representing more than 20 data points, are shown as mean ± SEM. Data have been compared by one-way ANOVA with Tukey test for multiple comparison. *P < 0.05, **P < 0.01, ***P < 0.001, ****P < 0.0001. (**c**) Tau:suramin condensates with heparin. Representative size distribution of Tau:suramin condensates at 25 μM Tau in low salt (10 mM NaCl) buffer. Time resolved DLS (trDLS) detects Tau monomers (radii ~ 7 to 10 nm) and mesoscopic Tau clusters (radii ~ 100 to 200 nm), the addition of 25 μM suramin after ~ 10 min induced the formation of Tau:suramin condensates with radii of 400–800 nm, at the cost of monomers and mesoscopic clusters in the solution. This was followed by the addition of 5 μM heparin after ~ 20 min, which results in the generation of smaller Tau:suramin condensates with radii of ~ 200 nm. Data point radius sizes correspond to DLS amplitudes, which are proportional to the intensity of scattered light of the respective particles. (**d**) Quantification of Tau:suramin condensate radii with heparin. Radii of condensates were calculated from 2 to 3 independent experiments, representing more than 20 data points are shown as mean ± SEM. Data have been compared by one-way ANOVA with Tukey test for multiple comparison. *P < 0.05, **P < 0.01, ***P < 0.001, ****P < 0.0001. (**e**) Brightfield microscopy of Tau:suramin, Tau:heparin, and Tau:heparin:suramin condensates (25 μM Tau, 0.014 mg/ml = ~0.8 μM heparin, 25 μM suramin in 25 mM HEPES, 10 mM NaCl, 1 mM DTT pH 7.4). Scale bar corresponds to 25 μm.

In Tau coacervates formed with heparin and RNA, oligomeric Tau species that can seed Tau aggregation in vitro and in cells can evolve over time^16^. Disrupting such Tau coacervates could therefore prevent the formation of seeding competent Tau and may open new therapeutic avenues for preventing pathological Tau aggregation. Tau coacervation occurs at charge matching conditions. We hypothesized that the addition of negatively charged suramin to Tau:heparin coacervates would disrupt the charge balance and thereby dissolve the coacervates. Indeed, the addition of 25 µM suramin immediately disrupted Tau:heparin coacervates (25 µM Tau, 5 µM heparin; before suramin: R_h_: ~ 500 nm) and monomeric Tau re-appeared (R_h_: ~ 15 nm; Fig. 3a,b). In a reverse experiment, in which we treated pre-formed Tau:suramin condensates with 5 µM heparin, most Tau:suramin condensates (before heparin: R_h_: ~ 500 nm) also dissolved into monomers (R_h_: ~ 10 nm; Fig. 3c,d). Light microscopy showed that Tau:heparin:suramin condensates, formed in the presence of both heparin and suramin, produced smaller condensates compared to Tau:heparin or Tau:suramin condensates (Fig. 3e). Comparable results were obtained with Tau:RNA coacervates (25 µM Tau, 50 μg/ml polyA RNA, R_h_: ~ 600 nm), where addition of 25 µM suramin dissolved Tau:polyA coacervates (after suramin: R_h_: ~ 15 nm; Supplemental Fig. S1c,d). Conversely, addition of RNA to pre-fomred Tau:suramin condensates (R_h_: ~ 1000 nm;) caused a shrinkage of the condensates at 50 μg/ml polyA (R_h_: ~ 600 nm; and dissolved them into smaller Tau species at 100 μg/ml polyA (R_h_: ~ 30 nm; Supplemental Fig. S1e,f).

In summary, we observed that Tau coacervates formed with polyanionic polymers can be disrupted by the addition of suramin, due to competition between polyanionic polymers and suramin to interact with positive charges on Tau.

### Suramin inhibits the formation of seeding competent Tau from Tau condensates

**Figure 4:**
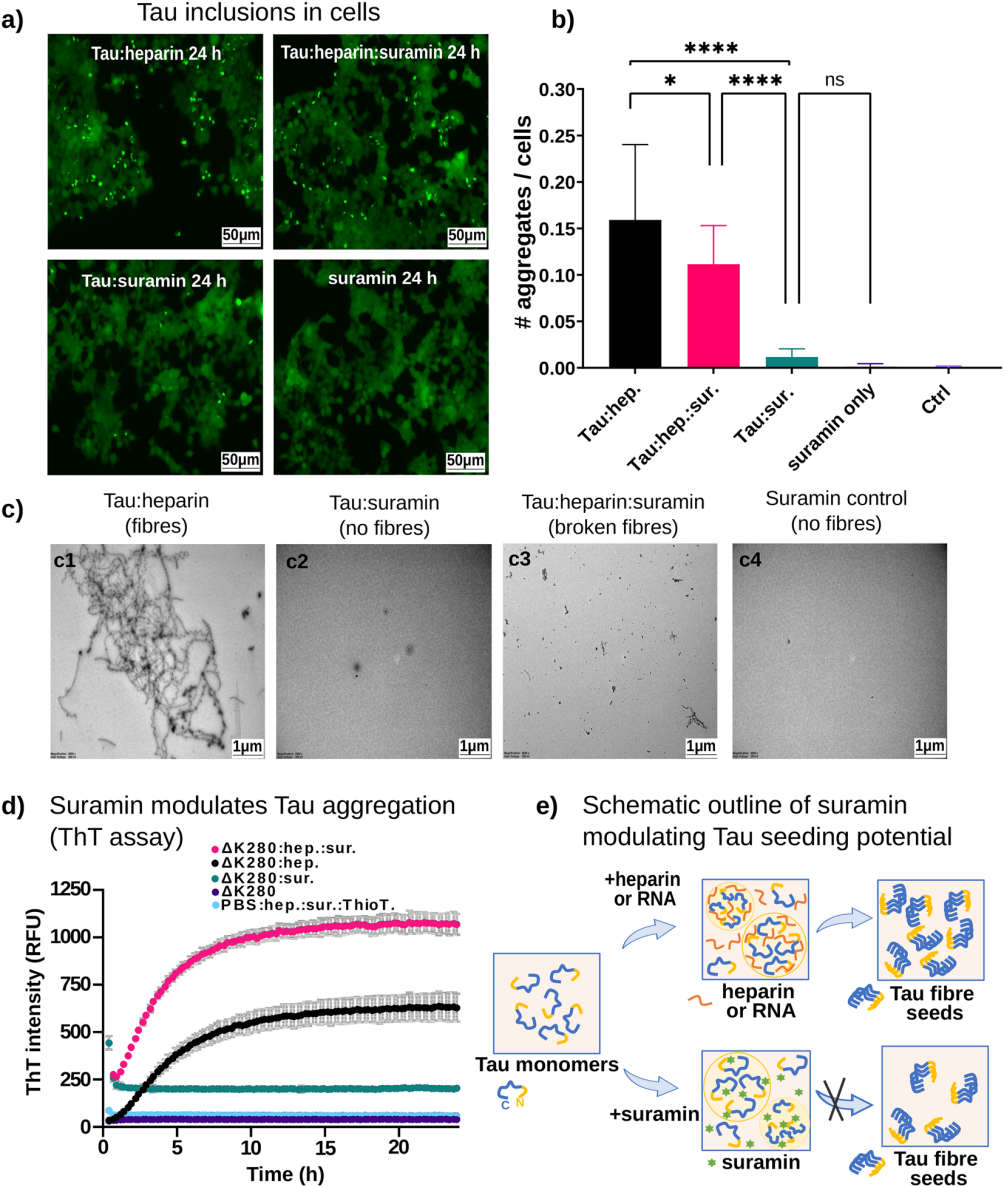
Tau:suramin condensates alone or in combination with heparin modulates seeding potential in cells. (**a**) Representative epifluorescence microscopy images of HEK sensor cells treated with 24 h-old Tau:heparin and Tau:suramin condensates. Tau incubated with heparin and suramin together (added to Tau at the same time) showed reduced seeding activity compared to Tau:heparin coacervates. Scale bar corresponds to 50 μm. (**b**) Quantification of aggregates per cell in cells treated with different Tau condensates. As a negative control (Ctrl) the non-treated cells were used. Data are shown as mean ± SD, three independent assays were analyzed. One-way ANOVA with Tukey post-test. Data have been compared by one-way ANOVA with Tukey test for multiple comparison. *P < 0.05, **P < 0.01, ***P < 0.001, ****P < 0.0001. (**c**) Suramin modulates Tau fibril formation in comparison to heparin. Representative transmission electron micrographs (TEM) of 36 h-old samples show full-length fibres (**c1**) for Tau:heparin condensates, versus broken Tau fibres (**c3**) for condensates incubated together with suramin and heparin, and no fibrils for (**c2**) Tau:suramin condensates or (**c4**) the suramin control. The concentrations used for Tau, heparin and suramin are 25 μM, 5 μM and 25 μM respectively. Scale bar corresponds to 1 μm. (**d**) ThioflavinT assay of Tau^ΔK280^ aggregation in the presence of heparin and suramin. Tau^ΔK280^ (10 μM) was mixed with heparin (2.5 μM) or suramin (10 μM), or heparin and suramin together (added at the same time) for 24 h in PBS with 1 mM DTT and ThioflavinT (50 μM). Tau^ΔK280^ without addition of heparin and PBS with heparin, suramin and ThioflavinT were used as negative controls. Data shown as mean ± SD, n = 3 technical replicates. (**e**) Schematic outine of Suramin modulating Tau seeding potential. Tau monomers form condensates with known inducers like heparin or RNA that results in the seeding potential of pathological Tau. Suramin forms Tau:suramin condensates but does not initiate the seeding potential of pathological Tau to develop fibrils. The scheme was drawn using Microsoft® PowerPoint® for Microsoft 365 MSO (Version 2211 Build 16.0.15831.20098) 64-bit.

Next, we investigated whether suramin could inhibit the formation of seeding competent Tau species in Tau coacervates, a process that may contribute to the initial formation of small Tau oligomers in the Tau aggregation cascade^21^. We used an established HEK Tau aggregation sensor cell line^67^, which expresses the Tau repeat domain (TauRD) with the pro-aggregant FTD-mutation P301S, C-terminally fused to CFP or YFP (TauRD^P301S^-CFP and/or YFP). In these cells, intracellular Tau accumulations can be initiated by the exposure of the cells to seeding competent Tau species in the cell culture medium. We previously used this model to show that seeding competent Tau species evolve in Tau coacervates^16^. Here, we analyzed the seeding potential of 24h-old Tau:heparin coacervates and compared it to the seeding potential of Tau:suramin and Tau:heparin:suramin condensates, in which heparin and suramin were added simultaneously to Tau (Fig. 4a,b). Surprisingly, the presence of suramin significantly reduced the amount of seeding in Tau:heparin coacervates, and Tau:suramin condensates did not have any seeding competence. This data shows that suramin is a repressor of Tau seed formation in Tau condensates, indicating that suramin can antagonize polyanionic polymer induced Tau conformations leading to beta-structure. Notably, when we added suramin to Tau:heparin coacervates pre-formed for 15min, no significant reduction in Tau seeding potential of the condensates was observed (Supplemental Fig. S2b,c). This observation suggests a fast irreversible evolution of Tau conformations in Tau:heparin condensates during the first hour of condensation that may lead to seeds. In summary, our observations indicate that suramin not only counteracts Tau:heparin coacervation but also modulates the seeding activity evolving from them. We do see that suramin can reduce Tau seeding when competing with heparin for Tau condensation (= addition of suramin and heparin at the same time). This condition produces significantly less seeding compared to Tau:heparin (Fig. 4a,b). However, when adding suramin to preformed Tau:heparin condensates (= addition of suramin 15 min after heparin), when LLPS has already happened, suramin has no effect on the seeding potential (Fig. S2b,c), likely because Tau:heparin interactions driving Tau seed formation are already established. Overall, our data highlight that Tau:suramin condensates do not produce seeding and therefore are the first Tau condensates known to not have pathological activity^16,21^. This finding suggests that other negatively charged compounds of low MW may also be used to disrupt the path from Tau condensation towards aggregation (Fig. 4e).

To further test whether suramin could prevent Tau aggregation into beta-structure containing fibrils, we performed TEM (Fig. 4c) and ThioflavinT (ThT) Tau aggregation assays (Fig. 4d), in which Tau aggregation is induced *in vitro* by heparin. Applying TEM we observed fibrillary Tau aggregates in Tau:heparin aggregation (incubation for 36 h at 37 °C), no fibrils in Tau:suramin incubations in the same conditions, and a few small fibrils in Tau:heparin:suramin incubations (Fig. 4c). Applying ThioflavinT assays (ThT), we found that heparin-induced aggregation of the pro-aggregant FTD-mutant Tau, Tau^ΔK280^, in PBS (Tau^ΔK280^:heparin)^16^ was not affected by the presence of suramin (Tau^ΔK280^:heparin:suramin), due to the extremely strong aggregation potential of this mutation. Interestingly, in the ThT assay data (Fig. 4d), in the presence of strongly pro-aggregant mutant Tau^ΔK280^ in PBS, we observed that suramin cannot halt Tau aggregation, although if added from the beginning on together with heparin. This suggests that suramin can inhibit Tau nucleation particular inside condensates, but does not act on Tau aggregation in conditions where Tau condensation is disabled, for example by a salt concentration.

By TEM analysis of wild type (WT) Tau aggregation under LLPS conditions showed long fibrils in the presence of heparin, which recapitulates previous results^40^ and in the presence of heparin and suramin many smaller fibril fragments instead of less but long fibrils were observed. It can be assumed that the total amount of aggregated Tau is similar for the TEM and ThT experiments performed. This explains why we do not see a difference between the presence and absence of suramin during Tau^ΔK280^:heparin aggregation in ThT assays. However, that the cellular seeding potential of condensates with suramin is reduced ultimately indicates that Tau aggregates produced under LLPS conditions may differ in their structure and seeding potential, regardless of the amount of Tau aggregation. An additional reason for the apparent discrepancy between TEM and ThT assays could be that the assays had different buffer compositions during the aggregation reaction: whereas Tau condensation with heparin and/or suramin is favored by HEPES buffer (in low salt) used for TEM, Tau aggregation is favored in PBS buffer used for ThT, as previously been reported by us^16^ and others^12^ indicating that LLPS and aggregation require different conditions. Notably, we used Tau^ΔK280^ and not WT in ThT assays because the Tau^ΔK280^ aggregation kinetics are much faster (~ 7 days for hTau40 vs. 6 h for Tau^ΔK280^), which allowed us to generate sufficient experimental replicates. Our experience and published data^12,16^ show that although the aggregation kinetics are very different between the two Tau versions, the effect of modulators is usually similar.

In the absence of heparin, however, suramin alone did not induce fibrillar Tau aggregation (Tau^ΔK280^:suramin) (Fig. 4d). Previously, the compounds baicalein^41^ (flavonoid, possessing partial negative charge)^42^ and phthalocyanine tetrasulfonate^43^ (negatively charged) showed a similar inhibition of Tau aggregation but have not attributed LLPS mediated pathway of interaction with Tau. Whether the presence of suramin or these compounds during Tau aggregation changes the structure of the emerging Tau fibrils is not known. A modulation of Tau fibril structure, examined by cryo-EM, was recently reported for Tau aggregation in the presence of NaCl, which resulted in the formation of Tau fibrils with a filled cavity at the core, like Tau fibrils isolated from chronic traumatic encephalopathy (CTE) brain^44^.

## Discussion

Our data show that suramin, a small molecule drug used in the treatment of parasitic diseases, has the capacity to induce Tau condensation, disrupts preformed Tau:polyanion coacervates, and prevents the formation of pathological seeding activity evolving in Tau coacervates. Suramin is a symmetric anionic molecule that contains two naphthalene-trisulfonic acid moieties resulting in six sulfonate groups that supply negative charges for the molecule. Suramin can interact with positively charged basic amino acids through the formation of ion pairs with spatially defined negatively charged groups^23,26,37,45,46^. For Tau, we observed that rather weak interactions (Kd ~ 17 μM) with suramin driven Tau condensation, similar to polyanionic polymer-induced Tau coacervation^8,16^. We speculate, that suramin, through multiple weak electrostatic interactions with positively charged residues in Tau, induces condensate formation, and via these short-range interactions, prevents the stabilization of hydrogen-bonds between and within beta-structure in the Tau repeat domain, which prevents Tau seed formation in condensates (Fig. 4e). Alternatively, cation-pi interactions, as reported for FUS phase separation^47^, between positively charged Tau residues and the double bond system in suramin could produce a similar effect.

Importantly, the intermolecular electrostatic interactions between Tau and suramin seem to outcompete electrostatic interactions of Tau with heparin and RNA in the condensed phase. In the cytosol, Tau can interact with numerous negatively charged biomolecules, including RNA^11,14^. To which extend these interactors can initiate Tau LLPS as part of a physiological Tau function is mostly unknown. So far, Tau’s canonical interaction partner tubulin was shown to induce Tau LLPS and co-condensate with Tau *in vitro*, leading to microtubule nucleation and bundling^16,48^. Whether Tau:tubulin co-condensation induced Tau aggregation as observed for Tau:RNA coacervates^11,15,16^, or produces Tau condensates that do not transition into seeding potent Tau, such as herein observed for Tau:suramin condensates, still needs to be evaluated.

We observed that suramin can act on the electrostatic interactions of Tau:RNA coacervates and thereby efficiently dissolve them into Tau monomers. This electrostatic competition between suramin and polyanionic polymers like RNA and heparin offers a unique possibility to interfere with the formation and stability of cellular Tau coacervates that have the potential to develop pathological activity. Further investigations applying other anionic small molecule compounds will show whether this mechanism can be employed to develop therapeutic approaches that interfere with Tau aggregation seeded by Tau condensates in the brain. Notably, it also needs to be made sure that (potential) functional Tau:RNA interactions are not hindered upon treatment with such compounds. Condensed Tau phases have also been implicated in the physiological binding of Tau to microtubules^29,49,50^ and it needs to be evaluated whether suramin (or similar compounds) would inhibit this interaction. It will be interesting to analyze, for example, the effect of compounds known to inhibit or modulate amyloid-like Tau aggregation, such as the green tea polyphenol (-)-epigallocatechin-3-gallate (EGCG)^51^, bis-ANS (4,4′-dianilino-1,1′-binaphthyl-5,5′-disulfonic acid), pthalocyanine tetrasulfonate (PcTS), Myricetin, Epigallocatechin or Baicalein (polyphenol)^41,43,52–56^, on the condensation of Tau, for example using DLS.

Previously, NMR and MST showed binding efficiency of Tau aggregation inhibitors to soluble Tau^35,57,58^. Our MST data showed moderate binding affinity (Kd ~ 17 μM) of suramin to Tau, supporting the establishment of weak multivalent electrostatic interactions as the driving force for Tau:suramin condensation. Strong molecular interactions^32–34^, for example formed between Tau and heparin (Kd ~ 1.2 μM)^57,59^, that immobilize Tau molecules and give beta-structural elements in the repeat domain time to arrange into stacks, could promote aggregation and fibril formation of otherwise highly soluble, unstructured Tau molecules. We postulate that candidate compounds, that allow or promote Tau condensation while inhibiting its aggregation could function and show moderate to weak interactions with Tau and thereby allow for structural flexibility and fluctuations in Tau molecules. Furthermore, like suramin, they should be small to reach charged residues in Tau, even in the condensed phase. Indeed, a recent study showed that the molecular structure and chemical nature of polyanions are important for their impact on Tau fibril formation. The same study also admits the lack in investigations of early interactions between polyanions and Tau, except for PEG or RNA in Tau phase separation^56^.

## Conclusion

Our results provide evidence that the dynamic exchange between polyanionic polymers (herein heparin and RNA) and anionic molecules (herein suramin) can modulate the phase separation of Tau. Beyond the manifold Tau PTMs (post translational modifications), this offers yet another possibility to tune Tau’s function based on the local cellular environment. Importantly, this information also inspires the search for modulators of Tau condensation, which disassemble Tau coacervates or prevent their transition into Tau seeds, as therapeutics prohibiting Tau aggregation in the brain^16,21^. We furthermore propose an analogy to the theory of *colloidal suspension stability*^60^: Minimal interactions between colloidal Tau particles (i.e., mesoscopic clusters) can trigger Tau precipitation (i.e., aggregation). In the presence of charged molecules (i.e., suramin), however, the colloidal Tau particles are kept in a state of dynamic floccules (i.e., Tau condensates). Inside dynamic Tau floccules (= condensates) having small polyanionic compounds (i.e., Tau:suramin condensates), Tau can maintain certain structural fluctuations and molecular diffusion. In contrast, inside floccules formed with polyanionic polymers (i.e., Tau:RNA and Tau:heparin coacervates), Tau molecules are arranged along the polymer, which compromises the orientation flexibility of Tau and enables Tau-Tau interactions that eventually leads to aggregation and fibril formation. We think that applying trDLS to study the effect of polyanionic compounds on protein condensation is well suited for investigating these processes.

## Material and methods

### Expression and purification of recombinant human full-length Tau

For the investigations we used recombinant human full-length wildtype Tau (hTau40; 441 aa; 2N4R isoform, UniprotKB P10636-8; Isoform Tau-F) expressed in *E. coli* BL21 Star (DE3) (Invitrogen) and purified by cation exchange column (HiTrap SP HP, 5 ml, GE Healthcare), as previously described^16,61^. The fractions containing purified Tau were pooled, concentrated using spin column concentrators (10–30 kDa MWCO, Thermo Fischer Scientific), and further purified through a size exclusion column (Superose 6 10/300, GE Healthcare). The fractions of purified monomeric Tau were concentrated in PBS with 1 mM DTT later and buffer exchanged prior to the experiments and stored in 25 mM HEPES, 10 mM NaCl, 1 mM DTT pH 7.4. The final Tau concentration was measured applying a BCA assay (BCA kit, PIERCE), and aliquots of purified tau were flash frozen and stored at – 80 °C for further use. The expression and purification methodology of pro-aggregant FTD-mutant Tau, Tau^ΔK280^ is the same as that of WT Tau.

### Tau condensate preparation for trDLS, light microscopy and TEM studies

To induce condensates, if not indicated differently final concentration of 25 µM suramin (Sigma-S2671; 1,429.17 g/mol, 1.4 kDa) and/or 0.85 to 5 µM heparin (Applichem; 8–25 kDa and MP Biomedicals-101931; 17–19 kDa) and/or 50 μg/ml polyA RNA (Sigma-P9403; MW 385.3) were added to 25 µM Tau diluted in 25 mM HEPES (pH 7.4), 10 mM NaCl, 1 mM DTT. All Tau condensates for DLS were prepared at room temperature and nuclease-free water was used for all buffers. The Tau condensates were imaged 1–2 h after formation by adding 2.5 µl of the solution onto an amine-treated glass-bottom dish (TC-treated Miltenyi, GC 1.5). The dish was closed and equipped with a ddH2O prewetted tissue at the inner edges to avoid evaporation. Imaging was performed on a widefield microscope (Eclipse-Ti, Nikon) using a 60x oil or 40x water objective. The rest of the samples were incubated at 37 °C for 24 h and used in the HEK sensor cell assay.

### Time-resolved dynamic light scattering (trDLS)

The trDLS experiments were performed with solutions of 25 µM Tau, 50 μg/ml to 100 μg/ml polyA, 0.85 µM or 5 µM heparin, and 25 to 100 µM suramin. The solutions were prepared at a two-fold concentration and centrifuged for 15 min at 16000 g at room temperature prior to trDLS experiments. To induce Tau LLPS, polyA or suramin were added to Tau in a DLS cuvette in concentrations mentioned above and trDLS measurements and data acquisition was carried out using a Spectroscatter-301 (Xtal Concepts, Germany) equipped with a laser providing a wavelength of 660 nm. Sample solutions were measured at a fixed 90° scattering angle in quartz-glass cuvettes (path length: 1.5 mm, Hellma Analytics, Müllheim, Germany) at 20 °C. The trDLS experiments were performed three times to confirm the reproducibility of the data. The obtained autocorrelation functions (ACFs) of each experiment were averaged over 20 seconds for each data point. Averaged ACFs were fitted by applying the CONTIN regularization software^62^, and corresponding hydrodynamic radii, R_h_, were calculated via the Stokes–Einstein equation,

$$R_{h} = \frac{{k_{B} T}}{6\pi \eta D}$$

with ***k***_***B***_ being the Boltzmann constant, ***T*** the temperature, ***η*** the viscosity, and ***D***_***t***_ the diffusion constant. The polydispersity index (PDI) is a measure of the size heterogeneity in a sample. Polydispersity can also occur due to agglomeration or aggregation in the sample. PDI values were calculated based on a nonlinear statistical method of the CONTIN software, where the maximum value for a monodispersed sample is 20%. The PDI for only monodispersed Tau before they form condensates are indicated at the onset of the DLS experiment. In short, PDIs were derived using the Xtal Concepts algorithm with the ${\mathbf{k}}_{{\mathbf{2}}} /\overline{\user2{\Gamma }}^{2}$ relationship, where ***Γ*** is the decay constant and is directly related to the diffusion behaviour of macromolecules (**Dτ**), whereas $\overline{\user2{\Gamma }}$ is the mean of ***Γ*** values and **k**_**2**_ is the variance of measured distributions for the decay rates of the Gaussian distribution^63,64^.

### Transmission electron microscopy (TEM)

For TEM experiments of Tau:suramin condensates (Fig. 1i), the Tau:suramin samples were prepared in the similar way as applied for DLS experiments in equimolar concentrations for Tau (25 µM) and suramin (25 µM) and 5 µM for heparin. About 3 µl of undiluted sample was loaded onto glow discharged carbon-coated copper grids, (Quantifoil R 1.2/1.3, Science Services), incubated for 30 seconds to allow adherence of condensates, blotted to remove excess solution, stained with 2% (w/v) uranyl acetate (UA) solution for 15 seconds and dried as per the standard negative staining protocol for proteins to reduce background and increase contrast. The morphology and dimensions of Tau:suramin condensates were analyzed by TEM (JEM-2100-Plus, JEOL, Germany) and micrographs were taken at an accelerating voltage of 200 kV. All TEM experiments were conducted in the XBI Biolab of European XFEL^65^. The experiments were repeated three times. For Tau TEM fibril investigations the Tau:suramin, Tau:heparin and Tau:suramin:heparin condensate samples were prepared and incubated at 37 °C for 36 hours along with suramin control (Fig. 4c).

### Small angle X-ray scattering (SAXS)

To confirm the sizes of monomer Tau, we performed SAXS experiments applying various concentrations of full-length Tau (hTau40). Since hTau40 is prone for aggregation, buffers with low (10 mM NaCl) and high salt (150 mM) concentrations were used in the measurements. Experiments were performed applying the EMBL beam line P12 (DESY/PETRAIII)^66^. Tau samples were centrifuged at 16,000 g for 15 minutes at 20°C prior to SAXS measurements.

### HEK sensor cell assay

HEK293 cells in 8-well (ibidi) and 96-well black cell culture microplates (Greiner), stably expressing the human 4R Tau repeat domain (TauRD) with the frontotemporal dementia (FTD)-mutation P301S and fused to CFP or YFP (HEK293 TauRD^P301S^-CFP/YFP^67^; ATCC #CRL-3275, cells provided by Marc Diamond through Erich Wanker), were treated with 24 h-old Tau condensate preparations (= 5 μg Tau) mixed with 0.8% lipofectamine 2000 (Invitrogen) in OPTI-MEM cell culture medium for 2 h at 37 °C in a total volume of 150 µl OPTI-MEM cell culture medium. After 2 h, the incubation mix was replaced against fresh culture medium, and cells were further incubated for 24 h at 37 °C. Induced intracellular TauRD^P301S^ accumulations were detected by microscopy using the YFP fluorescence in the green channel. Cells were imaged alive with a widefield fluorescence microscope (Eclipse Ti, Nikon) using a 20x air objective. The HEK sensor assay in Fig 4 and Fig S2 were performed in 8-well and 96-well plates respectively. Quantification of the number of Tau accumulations per number of cells (Hoechst = number of nuclei), was done in Image J.

### Microscale thermophoresis (MST) induced fluorescence

To measure the binding affinity of suramin with Tau in the condensates, we performed an MST experiment using Tau labelled with RED-MALEIMIDE 2nd Generation (Cysteine Reactive). In brief, Tau at 140 nM was co-incubated with suramin at different concentrations ranging from 153 nM to 5 mM and the fluorescence signal was recorded for around 20 s after laser-induced heating. The analysis of MST data and graph plots were prepared by Thermo Affinity online tool developed by eSPC facilty of EMBL^68^ and MO.Affinity Analysis v2.3 (Nano Temper) software provided by the manufacturer (Supplementary Fig. S2a).

### Fluorescence recovery after photobleaching (FRAP)

Tau:suramin condensates containing 2% DyLight-488 labeled Tau (labeled using amine-reactive DyLight488-NHS ester (Thermo Scientific) following manufacturer instructions) were imaged before and directly after bleaching with a 488 nm laser (90% intensity; 6 loops). The recovery of the fluorescence in the bleached region (circular ROIs, diameter 1 to 2 µm), a similar non-bleached reference-ROI (inside a different condensate) and a background ROI (Region of interest) were monitored in parallel for 40 s. FRAP curves were background corrected and normalized to the background corrected reference signal. Experiments were performed at room temperature on a spinning disk confocal microscope (Eclipse-Ti CSU-X, Nikon) using a 60x oil objective (Fig. 1g).

### ThioflavinT assay (ThT)

The change of ThioflavinT (50 μM, Sigma-Aldrich) fluorescence in the presences of Tau (10 µM Tau^ΔK280^) with heparin (0.041 mg/ml (2.5 µM), Applichem; 8–25 kDa) or suramin (10 µM; Sigma-S2671;  1,429.17 g/mol) in PBS containing 1 mM DTT was measured (at λ_Ex_ = 440 nm, λ_Em_ = 485 nm) in a plate reader (Infinite M Plex, Tecan) every 15 min after a 5 s shake at 37°C. Tau^ΔK280^ in PBS, 1 mM DTT without heparin and PBS in the presence of heparin (0.041 mg/ml) and suramin (10 µM) with Thioflavine-T (50 μM) were used as controls. The samples were prepared as triplicates in 384-well µClear plates (Greiner) (Fig. 4d).

### Data and statistical analysis

Image analysis was done applying ImageJ. Data plotting and statistical evaluation were performed using GraphPad Prism 8. Comparison of two groups was done by Student’s t-test, multiple groups were compared by one-way ANOVA with Tukey post-test as indicated in the figure legends. ****P < 0.0001, ***P < 0.001, **P < 0.01, *P < 0.05.

## Supplementary Information

Supplementary Figures.

## Data Availability

All data generated or analyzed during this study are included in this published article [and its supplementary information files].
