# Supplementary material for: Initiation and modulation of Tau protein phase separation by the drug suramin
Source: Sci Rep. 2023 Mar 9;13:3963. doi: 10.1038/s41598-023-29846-9 (PMC9997437; doi:10.1038/s41598-023-29846-9)
Supplement: Supplementary file 1 — Supplementary Figures. [file 41598_2023_29846_MOESM1_ESM.pdf]

Fig. S1

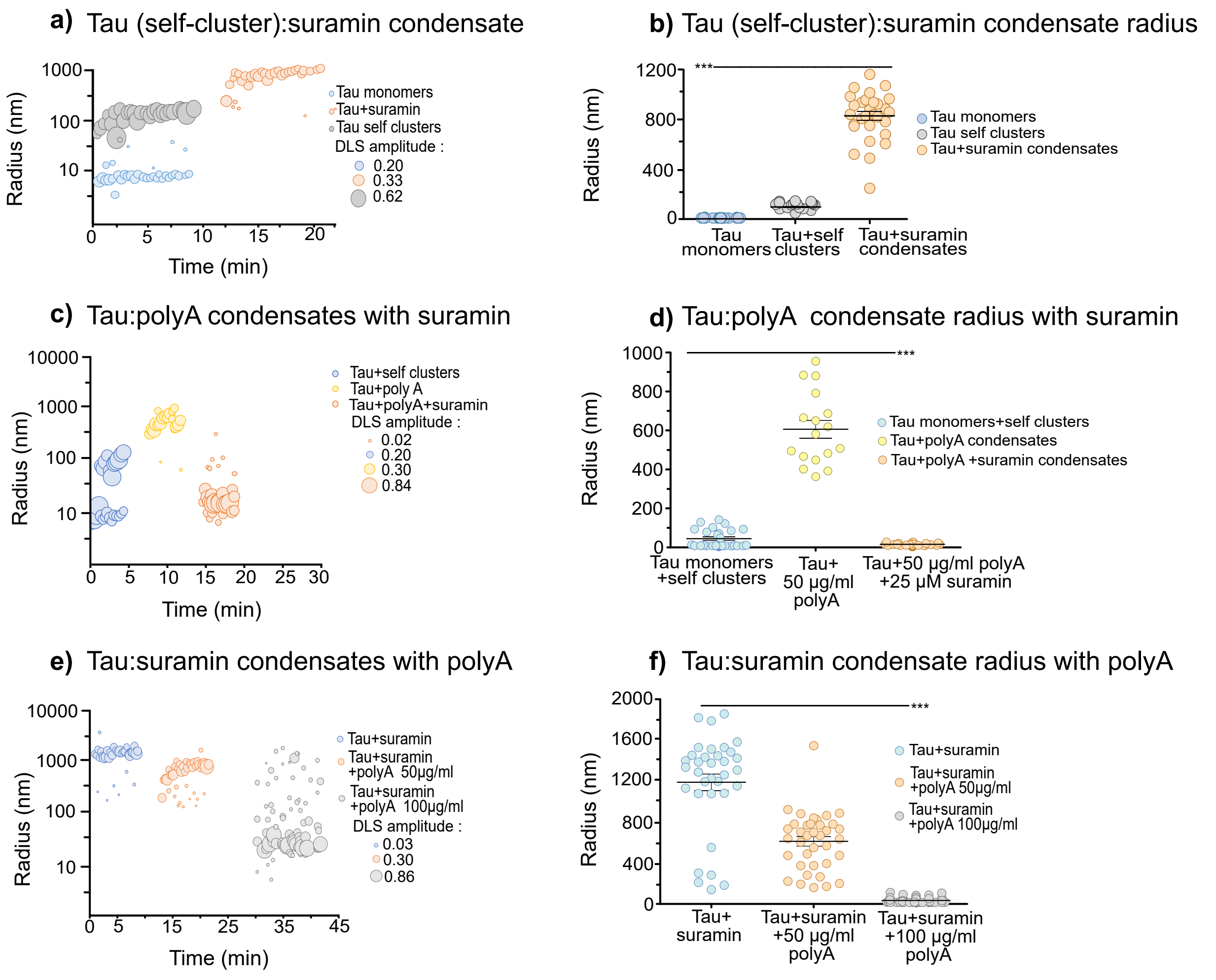
a) Tau (self-cluster):suramin condensate
Radius (nm)
1000
100
10
0
5
10
15
20
Time (min)
Tau monomers
Tau+suramin
Tau self clusters
DLS amplitude :
0.20
0.33
0.62
b) Tau (self-cluster):suramin condensate radius
Radius (nm)
1200
800
400
0
***
Tau monomers
Tau+self clusters
Tau+suramin condensates
Tau monomers
Tau self clusters
Tau+suramin condensates
c) Tau:polyA condensates with suramin
Radius (nm)
10000
1000
100
10
0
5
10
15
20
25
30
Time (min)
Tau+self clusters
Tau+poly A
Tau+polyA+suramin
DLS amplitude :
0.02
0.20
0.30
0.84
d) Tau:polyA condensate radius with suramin
Radius (nm)
1000
800
600
400
200
0
***
Tau monomers +self clusters
Tau+ 50 µg/ml polyA
Tau+50 µg/ml polyA +25 µM suramin
Tau monomers+self clusters
Tau+polyA condensates
Tau+polyA +suramin condensates
e) Tau:suramin condensates with polyA
Radius (nm)
10000
1000
100
10
0
5
15
25
35
45
Time (min)
Tau+suramin
Tau+suramin +polyA 50µg/ml
Tau+suramin +polyA 100µg/ml
DLS amplitude :
0.03
0.30
0.86
f) Tau:suramin condensate radius with polyA
Radius (nm)
2000
1600
1200
800
400
0
***
Tau+ suramin
Tau+suramin +50 µg/ml polyA
Tau+suramin +100 µg/ml polyA
Tau+suramin
Tau+suramin +polyA 50µg/ml
Tau+suramin +polyA 100µg/ml

Supplemental Fig. S1.

(a) Representative size distribution of Tau (self-cluster):suramin condensates : At 50 µM Tau in low salt (10 mM NaCl) buffer, trDLS detects Tau monomers (radii ~ 7 to 10 nm) and mesoscopic Tau clusters (radii ~100–200 nm). The addition of 25 µM suramin after ~7 min induced the formation of Tau:suramin condensates with radii of 400 - 1200 nm, at the cost of monomers and mesoscopic clusters in the solution. Data point radius sizes correspond to DLS amplitudes, which are proportional to the intensity of scattered light of the respective particles.

(b) Radius of Tau:suramin condensates calculated from 3 independant experiments and more than 20 data points of representative experiments are shown as mean ± SEM.

(c) Representative size distribution of Tau:polyA condensates with suramin : At 25 µM Tau in low salt (10 mM NaCl) buffer, trDLS detects Tau monomers (radii ~ 7 to 10 nm) and mesoscopic Tau clusters (radii ~100–200 nm). The addition of 50 µg/ml polyA RNA after 7 min induced the formation of Tau:polyA condensates with a radius of ~600nm, at the cost of monomers and mesoscopic clusters in the solution, followed by addition of 25 µM suramin (at ~15 min), which dissolve the condensates into monomers (~10 – 15 nm).

(d) Radius of Tau:polyA condensate upon addition of suramin calculated from 2 - 3 independant experiments and more than 20 data points of representative experiments are shown as mean ± SEM.

(e) Representative size distribution of Tau:suramin condensates with polyA : At 25 µM Tau in low salt (10 mM NaCl) buffer mixed with 25 µM suramin, trDLS detects preformed Tau:suramin condensates (radii ~ 800 to 1000 nm), with addition of 50 µg/ml polyA RNA (~after 10 min) a reduction in the size of condensates (~600nm) is detected, further addition of 100 µg/ml polyA (after ~35 min) caused a significant reduction of the radius (~30nm).

(f) Radius of Tau:suramin condensate with polyA calculated from 2 - 3 independent experiments and more than 20 data points from representative experiments are shown as mean ± SEM.

Data statistics: Data in "**b, d, f**" have been compared by one-way ANOVA with Tukey test for multiple comparison. *P < 0.05, **P < 0.01, ***P < 0.001, ****P < 0.0001.

Fig. S2

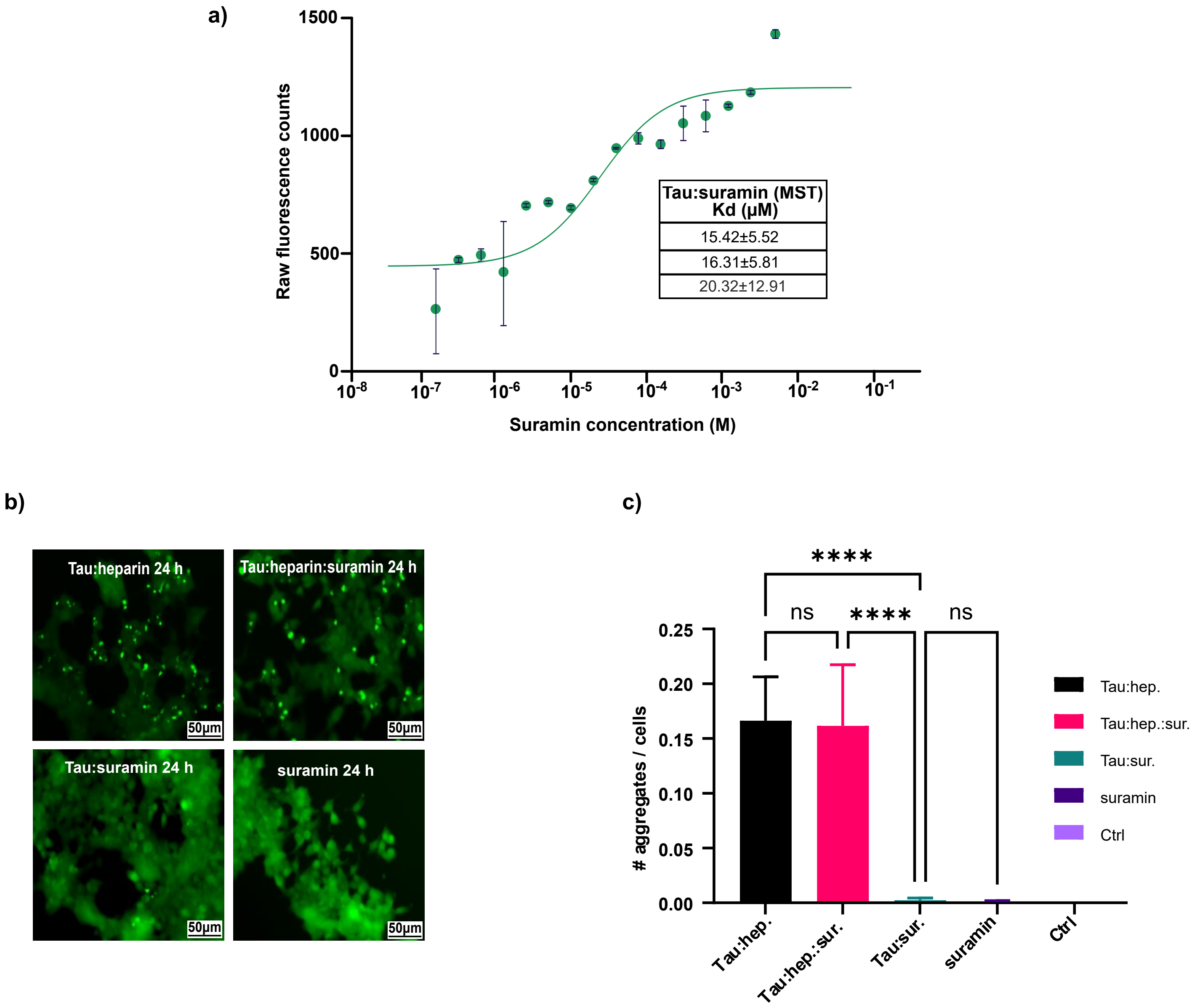
a)
Raw fluorescence counts
1500
1000
500
0
10-8
10-7
10-6
10-5
10-4
10-3
10-2
10-1
Suramin concentration (M)
Tau:suramin (MST) Kd (µM)
15.42±5.52
16.31±5.81
20.32±12.91
b)
Tau:heparin 24 h
Tau:heparin:suramin 24 h
Tau:suramin 24 h
suramin 24 h
50µm
50µm
50µm
50µm
c)
****
ns
****
ns
aggregates / cells
0.25
0.20
0.15
0.10
0.05
0.00
Tau:hep.
Tau:hep.:sur.
Tau:sur.
suramin
Ctrl
Tau:hep.
Tau:hep.:sur.
Tau:sur.
suramin
Ctrl

Supplemental Fig. S2.

(a) MST analysis of suramin interaction with Tau.

Microscale thermophoresis (MST) analysis was performed for Tau at 140nM with suramin as the binding ligand following a gradient concentration ranging from 5mM to 153 nM. The equilibrium dissociation constant, Kd value, monitoring the suramin interaction was calculated by fitting the initial fluorescence data against the gradient concentration. Individual data points represent the mean of normalized relative fluorescence against the concentration of suramin. The experimental data points are represented by green dots and the fitting by a green line. The resulting data were calculated from three independent experiments and plotted using MO.Affinity Analysis v2.3 (Nano Temper) software provided by the manufacturer and Thermo Affinity, eSPC, EMBL.

(b) Representative epifluorescence microscopy images of HEK sensor cells treated with 24h-old Tau:heparin and Tau:suramin condensates. Tau incubated with heparin and suramin (Suramin was added 15 min later). Scale bar corresponds to 50 μm.

(c) Quantification of aggregates per cell in cells treated with different Tau condensates, representative images shown in (b). Data are shown as mean±SD, three images per three technical replicates in three independent assays were analyzed. One-way ANOVA with Tukey post-test. Data have been compared by one-way ANOVA with Tukey test for multiple comparison. *P < 0.05, **P < 0.01, ***P < 0.001, ****P < 0.0001.
